# Prevertebral Abscess Revealing a Rare Foreign Body: A Case Report

**DOI:** 10.3390/jpm16070370

**Published:** 2026-07-09

**Authors:** Theresa Mally, Nina Rubicz, Paul Martin Zwittag

**Affiliations:** 1 Department of Otorhinolaryngology, Head and Neck Surgery, Kepler University Hospital, 4020 Linz, Austria; theresa.mally1999@gmail.com (T.M.); nina.rubicz@kepleruniklinikum.at (N.R.); 2 Medical Faculty, Johannes Kepler University Linz, 4020 Linz, Austria

**Keywords:** foreign body migration, prevertebral abscess, retropharyngeal abscess, dysphagia, computed tomography, deep neck infection, cervical surgery, case report

## Abstract

**Background:** Migrated foreign bodies in the prevertebral region represent a rare but potentially serious condition. This case underscores the importance of thorough visual and digital intraoperative exploration for successful foreign body retrieval and highlights the potential diagnostic and surgical challenges associated with migrated foreign bodies in the cervical region. **Case report:** A 77-year-old female patient presented with dysphagia following the intake of an Angiotensin-Converting Enzyme (ACE) inhibitor. Due to hemodynamic instability and laboratory findings that indicated multiple organ failure, a computed tomography (CT) scan was performed, which revealed a left-sided prevertebral abscess with gas collections. Because of persistently elevated and fluctuating inflammatory markers, multiple CT scans were performed, which showed an obliquely oriented, wire-like tubular structure approximately 30 mm in length, 5 mm in width and 1 mm in diameter in the prevertebral region of the previous abscess cavity. Eventually, after three surgical interventions—one transoral and two transcervical approaches—the foreign body could be identified and removed. Afterwards, the patient’s inflammatory markers decreased and her dysphagia resolved. **Conclusions:** This case demonstrates that early diagnosis and timely removal of foreign bodies in the cervical space are essential to prevent complications such as retropharyngeal abscess formation or mediastinitis. A combination of careful clinical examination, endoscopic evaluation, and cross-sectional imaging—particularly CT scan—is crucial for accurate localization. Finally, this report highlights the importance of maintaining a high index of suspicion for migrated foreign bodies in patients presenting with persistent symptoms or unexplained cervical infections following suspected foreign body ingestion.

## 1. Introduction

Foreign body ingestion is a rare but potentially serious emergency encountered in otorhinolaryngology practice, with fish bones representing the most frequently reported foreign bodies [1]. Patients typically present with acute throat discomfort, dysphagia, odynophagia, hoarseness, cough, or dyspnea, although some individuals may remain asymptomatic. In most cases, the foreign body can be identified and removed during physical examination or flexible and rigid endoscopy [1].

Occasionally, foreign bodies may penetrate the pharyngeal or esophageal mucosa and migrate into adjacent soft tissues or deep neck spaces. Such migration may result in serious complications, including cervical abscess formation, retropharyngeal abscess, parapharyngeal abscess, or mediastinitis, all of which may become life-threatening if diagnosis and treatment are delayed [1,2]. Retropharyngeal abscesses are relatively uncommon in adults, with an estimated incidence of 4.1 cases per 100,000 hospital admissions; however, their occurrence has increased over the past two decades [3].

When clinical examination and endoscopic evaluation fail to identify a suspected foreign body, imaging plays a pivotal role in the diagnostic workup. Computed tomography (CT) is considered the imaging modality of choice for detecting migrated foreign bodies, accurately defining their anatomical location, and assessing associated complications [1,2]. In patients presenting with severe symptoms, deep neck infection, or suspected migration, prompt surgical exploration may be required to prevent further morbidity [4].

We report a rare case of a migrated transparent plastic foreign body located in the prevertebral space that presented with a cervical abscess and persistent inflammatory activity. The case highlights the diagnostic challenges associated with migrated foreign bodies, the importance of repeated imaging and multidisciplinary management, and the role of meticulous visual and digital intraoperative exploration in achieving successful foreign body retrieval.

## 2. Case Presentation

A 77-year-old female patient presented to an outpatient clinic with dysphagia following the intake of an ACE inhibitor (Figure 1). The initial working diagnosis was angioedema secondary to ACE inhibitor use. The patient was admitted to the ward for treatment of the edema with corticosteroids and for further observation. However, ACE inhibitor–associated angioedema is a bradykinin-mediated condition and is typically not accompanied by elevated inflammatory markers or signs of systemic infection. The presence of elevated inflammatory parameters after submission was therefore not consistent with the initial working diagnosis. Consequently, empirical antibiotic therapy with ampicillin/sulbactam was initiated.

At admission, the patient was hemodynamically and respiratory stable and the videolaryngoscopy findings were unremarkable, as was the remainder of the otorhinolaryngological examination; however, two days later, she developed hemodynamic instability, and laboratory findings indicated multiple organ failure.

Based on this clinical deterioration, the patient was transferred to the intensive care unit, where she was intubated, required vasopressor support and became catecholamine-dependent. A CT scan was performed, which revealed a left-sided prevertebral abscess with gas collections. In response to these findings and the complex cervical abscess, antimicrobial therapy was escalated from ampicillin/sulbactam to meropenem to provide broader empiric coverage for severe deep neck infection and potential polymicrobial sepsis.

After the patient’s condition stabilized, a follow-up CT scan was performed. This scan revealed a foreign body located ventral to the fifth cervical vertebra, measuring 30 mm in length (Figure 2).

It was assumed that the foreign body had perforated the esophageal wall and subsequently migrated to a ventral position at the level of the fifth cervical vertebra. Comprehensive otolaryngologic examination and esophagogastroscopy revealed no swelling, mucosal defects, or other signs of injury in the laryngeal or hypopharyngeal region or within the esophagus. To exclude esophageal perforation as the potential route of entry, esophagogastroscopy was performed. However, no mucosal defects, signs of perforation, or other abnormalities were detected. Furthermore, the patient did not recall any history suggestive of foreign body ingestion, and there were no external signs of trauma or injury to the neck that could have explained direct penetration from outside.

Following interdisciplinary consultation with the cardiothoracic surgery department, due to the CT findings, mediastinitis was considered unlikely and subsequently ruled out. Following consultation with Kepler University Hospital, the patient was transferred for further surgical management and removal of the foreign body. During transfer, the patient was hemodynamically stable, still intubated and catecholamine-independent. At the university hospital, the patient underwent panendoscopy for removal of the foreign body.

Due to its lower morbidity and mortality, the transoral approach is generally considered the preferred surgical treatment. However, in cases of extensive deep neck infection, a transcervical approach may be required, as it provides improved exposure and a wider operative field. Both approaches are established and widely accepted techniques for the management of parapharyngeal and prevertebral abscesses [3,5].

In our case, an incision was made dorsal to the left piriform recess, resulting in a small discharge of pus. The incision was extended deep to the posterior pharyngeal wall, thereby opening the abscess cavity. The cavity was repeatedly irrigated and pus was aspirated; however, the foreign body could not be identified. Even intraoperative X-ray imaging for foreign body identification was omitted due to weak opacification in the CT scan, as a clear differentiation of the foreign body from the tissue was not expected. Afterwards, the patient was stable enough to be extubated and transferred to the general ward.

Following the procedure, the patient remained clinically stable, and physical examination findings remained unremarkable. Because of the persistent deep neck infection and concern for resistant Gram-positive pathogens, linezolid was added to the antimicrobial regimen. Due to the unsatisfactory outcome of the transoral approach and the persistent suspicion of a retained foreign body, as well as elevated inflammatory markers despite antibiotic treatment with meropenem, a second surgical procedure was performed. During this procedure, a transcervical approach to the left side of the neck was undertaken. Despite thorough digital and visual exploration, no foreign body could be located. The surgical site was closed, and as inflammatory markers decreased and no evidence of methicillin-resistant Staphylococcus aureus (MRSA) or other resistant Gram-positive pathogens emerged, linezolid was discontinued while meropenem therapy was continued.

Furthermore, two sets of blood cultures were collected during episodes of increasing inflammatory markers. Despite persistent signs of infection, no bacterial growth was detected, possibly due to ongoing broad-spectrum antibiotic therapy.

During postoperative clinical follow-up, the left lateral wall of the pharynx appeared edematous and swollen. After consultation with the radiology department, the possibility of a prevertebral bone fragment, which may have perforated the esophagus and subsequently migrated into the prevertebral space, was considered. Based on these findings and after interdisciplinary discussion, a conservative management approach with continued meropenem therapy was chosen.

Due to persistently elevated and fluctuating inflammatory markers under meropenem therapy (CRP 9.7 mg/dL), another CT scan was performed (Figure 3). The follow-up CT scan continued to show a radiopaque, linearly configured foreign body measuring approximately 25 mm in length in the prevertebral space at the level of C7/Th1. Laboratory tests demonstrated leukocyte counts within the normal range, whereas C-reactive protein (CRP) remained markedly elevated at 24.4 mg/dL. The clinical ENT examination was still unremarkable.

Because of this renewed increase in CRP levels, another CT scan was performed to identify the focus of infection. This examination revealed an obliquely oriented, wire-like tubular structure approximately 30 × 5 × 1 mm (length × width × thickness) in the prevertebral region of the previous abscess cavity (Figure 4). An additional a whole-body PET/CT was performed to exclude other potential inflammatory foci. The examination revealed focal increased FDG uptake confined to the prevertebral region corresponding to the known retained foreign body, without evidence of additional sites of infection or inflammation.

Based on the imaging findings with no evidence of an alternative inflammatory focus in the whole body and persistently elevated inflammatory markers despite ongoing antibiotic therapy, a repeat transcervical exploration of the left side of the neck was performed. During this procedure, the foreign body was ultimately identified without the help of intraoperative imaging and only by meticulous digital palpation of the inflamed soft tissue and subsequently removed using forceps. The surgical team identified a transparent, rigid plastic fragment measuring approximately 30 × 5 × 1–2 mm (length × width × thickness), with sharp and pointed edges (Figure 5).

Notably, no intraoperative microbiological specimens, including pus swabs or tissue cultures, were collected during any of the surgical interventions. As the patient had already received prolonged broad-spectrum antibiotic therapy before each intervention, the diagnostic yield of intraoperative cultures was considered to be limited.

Following removal of the foreign body, the patient’s inflammatory markers decreased, her dysphagia resolved, and she was discharged home in improved general condition (Figure 6). No scheduled outpatient follow-up was performed; therefore, long-term recurrence and late complications could not be formally assessed.

## 3. Discussion

Previous reports have demonstrated that migrated foreign bodies may penetrate the pharyngeal or esophageal wall and subsequently migrate into the retropharyngeal or parapharyngeal spaces, resulting in deep neck infections, abscess formation, and, in severe cases, mediastinitis [1,2,4,6]. Chen et al. analyzed 20 cases of migratory pharyngeal foreign bodies and highlighted the diagnostic challenges associated with delayed presentation and migration beyond the upper aerodigestive tract [1]. Similarly, Wang and Ma reported a migrated hypopharyngeal foreign body that progressed into the parapharyngeal space, emphasizing the importance of repeated imaging and careful surgical planning [2].

In the present case, the exact mechanism by which the foreign body reached the prevertebral space remains uncertain. However, the most plausible explanation is inadvertent ingestion of the foreign body, followed by penetration of the pharyngeal or esophageal mucosa and subsequent migration into the prevertebral soft tissues. Notably, the patient did not recall any episode of foreign body ingestion, and no mucosal defect was identified on endoscopic/gastroscopic examination. Furthermore, there were no external signs of trauma or injury to the neck that could have explained direct penetration from outside. Therefore, although the precise route of entry cannot be definitively established, transoral ingestion with secondary migration through the upper aerodigestive tract appears to be the most likely mechanism. But the presumed transoral route of entry remains speculative and should be interpreted with caution.

A thorough otolaryngologic examination, including laryngoscopy, is essential to identify, localize, and remove the foreign body. However, negative findings on endoscopic examination do not exclude the presence of a migrated foreign body, particularly when the object has already penetrated the mucosa. In such cases, imaging modalities play a crucial role in the diagnostic workup. CT is considered the imaging modality of choice for detecting migrated foreign bodies, accurately defining their anatomical location, and assessing their relationship to adjacent structures and associated complications [6,7].

Although the retrieved foreign body was macroscopically transparent, it remained detectable on serial CT examinations. This may be attributable to the attenuation characteristics of the plastic material itself as well as to the surrounding inflammatory reaction and abscess formation, which increased the conspicuity of the foreign body and its associated soft-tissue changes on CT imaging [8].

The educational value of this case lies in the unusual nature of the foreign body and the resulting diagnostic difficulties. In contrast to the majority of published cases, which involve fish bones or other radiopaque foreign bodies, the retrieved object consisted of a thin, transparent plastic fragment [1,2,4,6]. Reports describing migrated plastic foreign bodies within the prevertebral space are exceedingly rare. The transparent appearance of the foreign body, combined with its small size and deep location within inflamed tissue, substantially complicated both radiological interpretation and intraoperative identification.

If a foreign body remains undetected, local infection may develop, potentially leading to a retropharyngeal abscess, which can present with highly variable clinical manifestations and may complicate subsequent surgical removal [4]. In cases of persistent symptoms or unexplained inflammatory markers, surgical exploration should be considered even when initial endoscopic findings are inconclusive. With regard to surgical management, both transcervical and endoscopic approaches may be considered depending on the localization of the foreign body and the complexity of the prevertebral abscess [3,4,5]. Successful removal primarily depends on precise preoperative localization [4]. In the present case, both identification and removal of the foreign body proved particularly challenging during the initial surgical interventions.

When a retropharyngeal abscess is suspected, prompt initiation of broad-spectrum antibiotic therapy is essential in order to prevent the spread of infection into the mediastinum [4]. Acute mediastinitis represents a severe infectious complication associated with a reported mortality rate of 20–40% and therefore constitutes a life-threatening condition [4].

This case illustrates the considerable diagnostic difficulty posed by migrated foreign bodies in the cervical space, particularly when surrounded by inflamed tissue and purulent collections. Multiple CT scans and three surgical procedures were ultimately required to locate and remove the foreign body. During the first two surgical explorations, large amounts of purulent material were evacuated; however, despite meticulous visual and digital intraoperative exploration, the foreign body could not be identified. It was finally detected and removed during the third procedure. The retrieved object was an extremely thin, pointed, and transparent plastic fragment. These characteristics likely explain the considerable difficulty in both detection and retrieval.

Compared with previously reported cases of migrated foreign bodies causing retropharyngeal or parapharyngeal abscesses, the present case is distinguished by the combination of a transparent plastic foreign body, the absence of a documented ingestion event, repeated unsuccessful surgical explorations, and prolonged persistence despite extensive antimicrobial therapy [1,2,4,6]. These features highlight the importance of maintaining a high index of suspicion and considering retained foreign material even when clinical history and endoscopic findings are inconclusive.

## 4. Conclusions

In conclusion, migrated foreign bodies in the prevertebral region represent a rare but potentially serious condition. Early diagnosis and timely removal are essential to prevent complications such as retropharyngeal abscess formation or mediastinitis. A combination of careful clinical examination, endoscopic evaluation, and cross-sectional imaging—particularly CT scans—is crucial for accurate localization of the foreign body. When imaging findings remain suggestive despite inconclusive endoscopic results, surgical exploration may be required to ensure definitive removal and to prevent persistent infection. If no foreign body can be found, the surrounding soft tissues should be thoroughly examined by careful digital palpation during surgery as transparent plastic foreign bodies can also be present, as in our case.

## Figures and Tables

**Figure 1:**
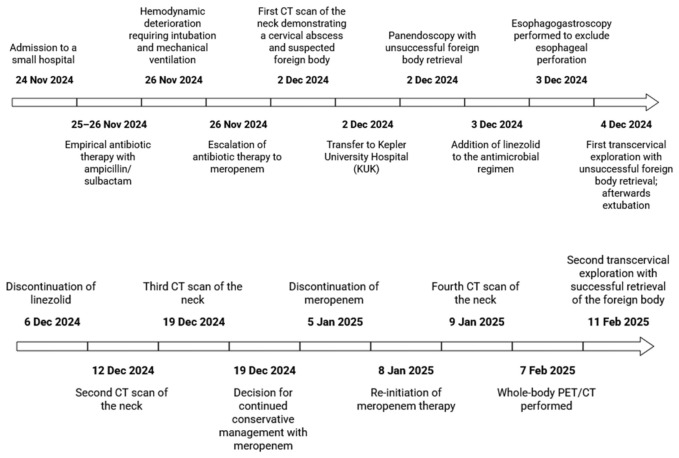
Timeline of events.

**Figure 2:**
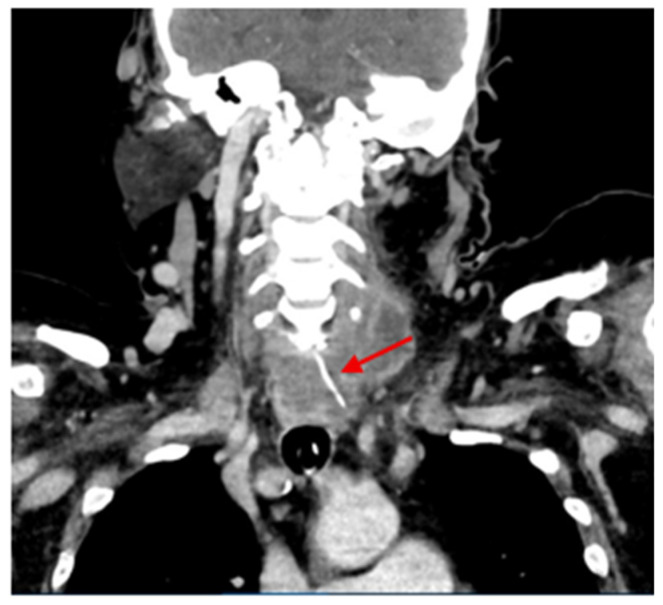
Initial contrast-enhanced CT scan demonstrating a linear foreign body in the prevertebral region at the level of C5.

**Figure 3:**
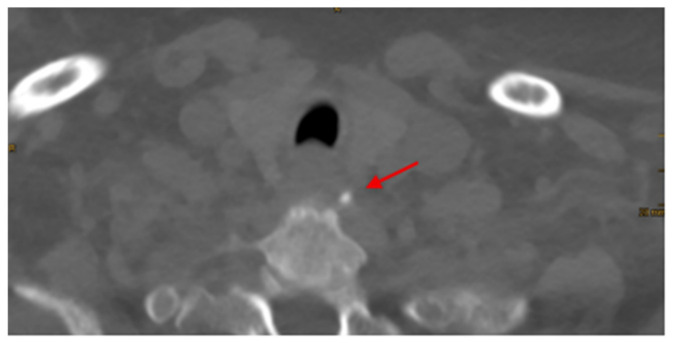

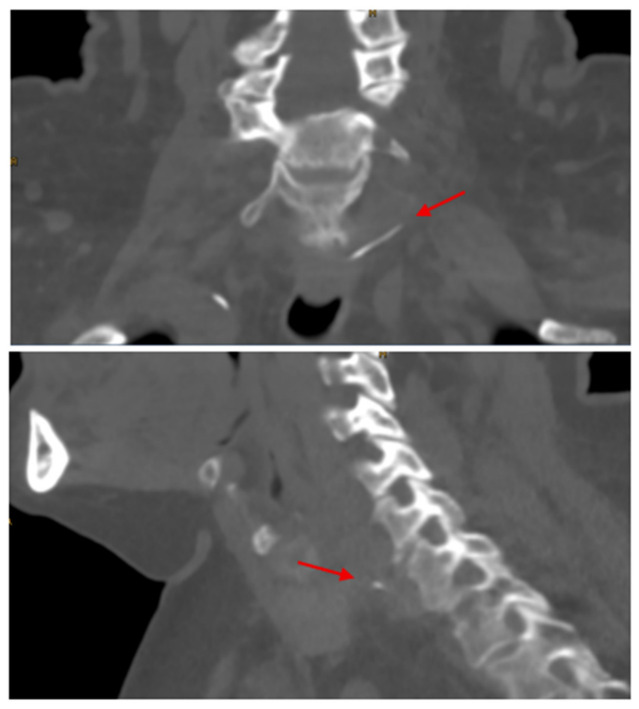
Follow-up CT scan showing persistence of the linear foreign body within the prevertebral space despite previous surgical exploration.

**Figure 4:**
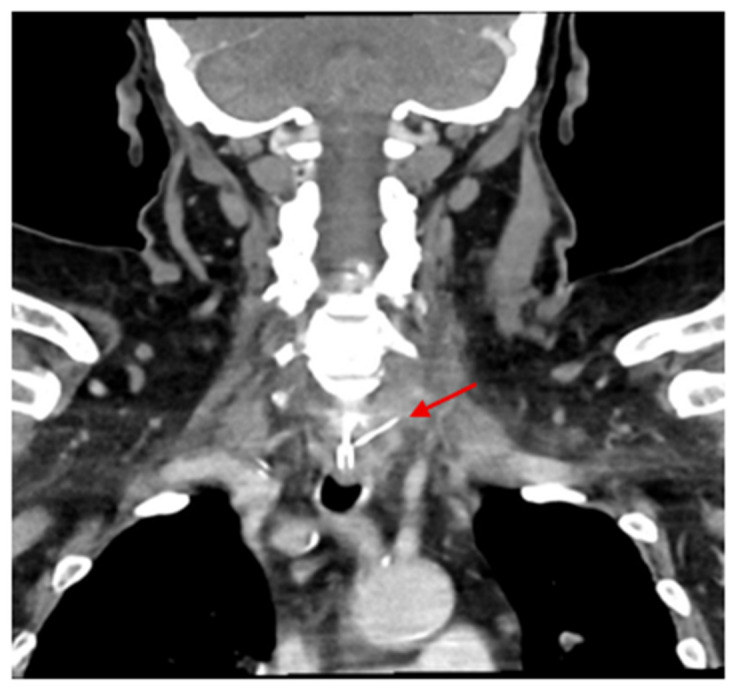
Contrast-enhanced CT scan obtained before the third surgical intervention showing the retained foreign body.

**Figure 5:**
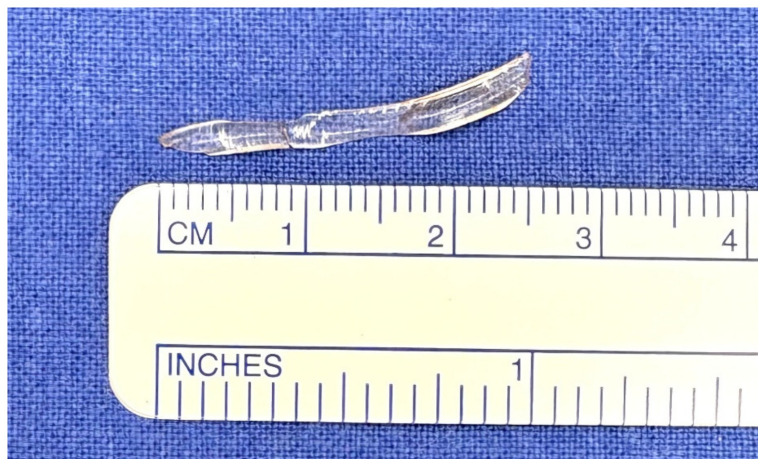
Transparent plastic foreign body removed during the third transcervical exploration. The fragment measured approximately 30 mm × 5 mm × 1–2 mm and exhibited sharp, pointed edges.

**Figure 6:**
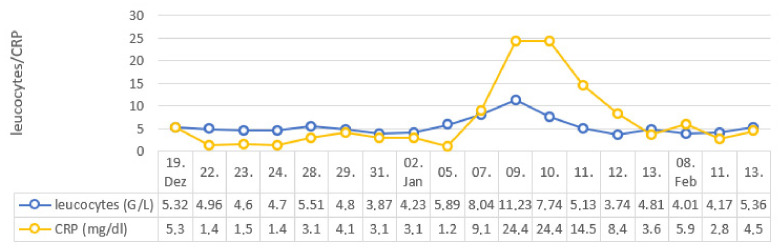
Evolution of inflammatory markers. Leucocytes G/L, normal range 4.4–9.7. CRP mg/dL, normal range 0.0–0.5.

## Data Availability

Data supporting the findings of this study are available from the corresponding author upon reasonable request.

## Abbreviations

The following abbreviations are used in this manuscript:

ACE	Angiotensin-Converting Enzyme
CT	computed tomography
